# A Remaining Useful Life Prognosis of Turbofan Engine Using Temporal and Spatial Feature Fusion

**DOI:** 10.3390/s21020418

**Published:** 2021-01-08

**Authors:** Cheng Peng, Yufeng Chen, Qing Chen, Zhaohui Tang, Lingling Li, Weihua Gui

**Affiliations:** 1 School of Computer, Hunan University of Technology, Zhuzhou 412007, China; chengpeng@csu.edu.cn (C.P.); yfengchen1698@hut.edu.cn (Y.C.); qinchen1228@hut.edu.cn (Q.C.); Lingli@hut.edu.cn (L.L.); 2 School of Automation, Central South University, Changsha 410083, China; whgui@csu.edu.cn

**Keywords:** remaining useful life (RUL), long short-term memory (LSTM), one-dimensional convolutional neural networks with full convolutional layer (1-FCLCNN), temporal and spatial features, turbofan engine

## Abstract

The prognosis of the remaining useful life (RUL) of turbofan engine provides an important basis for predictive maintenance and remanufacturing, and plays a major role in reducing failure rate and maintenance costs. The main problem of traditional methods based on the single neural network of shallow machine learning is the RUL prognosis based on single feature extraction, and the prediction accuracy is generally not high, a method for predicting RUL based on the combination of one-dimensional convolutional neural networks with full convolutional layer (1-FCLCNN) and long short-term memory (LSTM) is proposed. In this method, LSTM and 1- FCLCNN are adopted to extract temporal and spatial features of FD001 andFD003 datasets generated by turbofan engine respectively. The fusion of these two kinds of features is for the input of the next convolutional neural networks (CNN) to obtain the target RUL. Compared with the currently popular RUL prediction models, the results show that the model proposed has higher prediction accuracy than other models in RUL prediction. The final evaluation index also shows the effectiveness and superiority of the model.

## 1. Introduction

Turbofan engine is a highly complex and precise thermal machinery, which is the “heart” of the aircraft. About 60% of the total faults of the aircraft are related to the turbofan engine [1]. The RUL prediction of the turbofan engine will provide an important basis for predictive maintenance and pre maintenance. In recent years, because of the rapid development of machine learning and deep learning, the intelligence and work efficiency of turbofan engines have been greatly improved. However, as large-scale precision equipment, its operation process cannot be separated from the comprehensive influence of internal factors such as physical and electrical characteristics and external factors such as temperature and humidity [2]. The performance degradation process also shows temporal and spatial characteristics [3], which provide necessary data support and bring challenges for the RUL prediction of the turbofan engine. At the same time, the data generated by the operation process of turbofan engine have the characteristics of nonlinearity [4], time-varying [5], large scale and high dimension [6], which results in the failure of effective feature extraction, and the non-linear relationship between the extracted features and the RUL cannot be mapped, which are the key problems to be solved urgently.

Many models have been developed to predict the RUL of turbofan engines. Ahmadzadeh et al. [7] divided the predicting methods into four categories, including experimental, physics-based, data driven, and hybrid methods. Experimental type relies on prior knowledge and historical data, but the operating conditions and operating environment of the equipment are uncertain, which leads to large prediction accuracy error, and cannot be promoted in complex scenarios. The physical model uses the physical and electrical characteristics of the equipment to construct accurate mathematical equations to describe the degradation law of the equipment and predict its remaining life. Usually, it is difficult to obtain the physical model for large precision equipment such as turbofan engines, and its application is restricted. Data driven is independent of the failure mechanism of equipment, its key is to monitor and extract effective performance degradation data. The method lacks an analysis of the uncertainty of the predicted results, and a large amount of historical data is needed to build a high-precision model. Hybrid model is a new prediction method which combines two or more neural network models, which has become the mainstream research trend. Among them, the hybrid model composed of CNN [8,9,10] and LSTM is the most common one in the field of RUL prediction of turbofan engine. CNN has a strong feature extraction ability, which cannot only extract local abstract features, but also process the data with multiple working conditions and multiple faults [11,12,13], especially the one-dimensional CNN can be well applied to the time series analysis generated by sensors (such as gyroscope or accelerometer data [14,15,16]). It can also be used to analyze signal with fixed length period (such as audio signal). Zhang et al. [17] adopted a fully convolutional neural network for feature self-learning and reduced training parameters; a weighted average method was used to denoise the prediction results, and the bearing-accelerated life experiment verified the effectiveness of the proposed method. Yang et al. [18] proposed an intelligent RUL prediction method based on the dual CNN model architecture to predict the turbofan engine RUL, the first CNN model determines the initial failure point, and the second CNN model is used for RUL prediction. This method does not require any feature extractor. The original vibration signal can be received, and useful information can be retained as much as possible. The prediction results and evaluation indicators prove the effectiveness and superiority of the method. Hsu [19] applied several deep learning methods to assess the status of aircraft engines in operation, and to classify the stages of operational degradation so as to predict the functional remaining lifespan of components. Li et al. [20] designed a new data-driven method using deep convolutional neural network (DCNN) for prediction, time windows are used for sample preparation to better extract features, and experiments based on the C-MAPSS data set have confirmed the effectiveness of this method. In fact, for the turbofan engine dataset (C-MAPSS), recent studies have used CNN to extract its features and achieved good results, while one-dimensional CNN can extract sensor data from the dataset and the full convolutional layer can reduce training parameters and weights. 

Long short-term memory network (LSTM) is a kind of time recurrent neural network (RNN), which can solve the problems of gradient disappearance and gradient explosion in RNN. For LSTM, the special "three gate structure" enables it to capture a long range of dependence and process time series data. While, the RUL prediction of turbofan engine needs to process the time series data, and LSTM can obtain the optimal features of the time series data generated by the turbofan engine, and can also mine rules in time series. Zhang et al. [21] applied a method based on LSTM, which is specifically used to discover the underlying patterns embedded in the time series, so as to track the system performance degradation, thereby predicting RUL. Kong et al. [22] utilized polynomial regression to obtain health indicators, and then combined them with CNN and LSTM neural networks to extract spatiotemporal features. Song et al. [23] proposed a hybrid health prediction model that combines the advantages of the autoencoder neural network and the bidirectional long-term short-term memory (BLSTM) neural network, using the autoencoder as a feature extraction tool, and the BLSTM captures the characteristics of the bidirectional long-range dependence of features. The above methods are all tested on the C-MAPSS data set to verify the effectiveness and accuracy; however, there are common problems such as complex training process and low prediction accuracy.

We found that the data set of turbofan engine is composed of multiple time series, the data in different data sets contain different noise levels, so it is necessary to normalize the original data, which will eliminate the influence of noise, and realize data centralization to enhance the generalization ability of the model. At the same time, it is difficult to capture multi fault mode and multi-dimensional feature data in different operating environments. It is also necessary to use multi scene and multi time point data to extract effective features to improve prediction accuracy, traditional methods cannot extract temporal and spatial features simultaneously and effectively fuse them. In addition, single neural network model is difficult to extract enough effective information in the face of multiple working conditions and multiple types of features. 

The main contributions of this paper include: (1) use LSTM to extract the temporal characteristics of the data sequence, and learn how to model the sequence according to the target RUL to provide accurate results. (2) A one-dimensional full-convolutional layer neural network is adopted to extract spatial features, and through dimensionality reduction processing, the parameters and computational complexity of the training process are greatly reduced. (3) The spatiotemporal features extracted by the two models are fused and used as the input of the one-dimensional convolutional neural network for secondary processing. Comparing this method with other mainstream RUL prediction methods, the score and error control of the method proposed in this article are better than others, which proves the feasibility and effectiveness of this method. 

The rest of this article is arranged as follows: Part 2 is the basic theory, mainly introducing the model structure of neural network and evaluation indicators. The third part is the focus of this article, mainly including the proposed model structure, algorithm, training process, implementation flow. The fourth part is the experiment and result analysis, and the last part is summary and prospect.

## 2. Basic Theory

### 2.1. Convolutional Neural Network 

**Figure 1:**
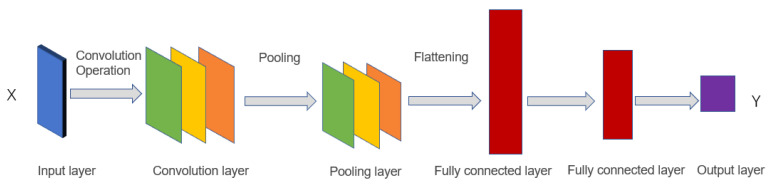
Traditional convolutional neural network.

Convolutional neural network has been widely used in image recognition, complex data processing [24]. The convolutional neural network consists of the input layer, convolutional layer, pooling layer, full connection layer, and output layer. Its basic components are shown in Figure 1.

The convolutional layer includes convolution kernel, convolutional layer parameters, and activation function. The main function is to extract features from the input data. Each element in convolution kernel has its own weight coefficient and bias. The convolution kernel regularly scans the input features, and performs matrix element multiplication and summation on the input features in the receptive field and superimposes the deviation [25], the core step is to solve the error term. To solve the error term, we first need to analyze which node needs to be calculated and which node or nodes in the next layer are related, because the node affects the final output result through the neurons connected with the node in the next layer, which also needs to save the relationship between each layer node and the node in the previous layer.

(1) $$\{\begin{array}{c} F=[A^{n}⊗w^{n+1}](i,j)=\sum_{m=1}^{Mn}\sum_{x=1}^{f}\sum_{y=1}^{f}\left[A_{m}^{n}(t*i+x,t*j+y)w_{m}^{n+1}(x,y)\right]\\ A^{n+1}(i,j)=F(i,j)+d \end{array}$$

(2) $$N_{n+1}=\frac{N_{n}+2P-f}{t}+1$$

In the formula, i, j are pixel indexes, d  is the bias in the calculation process, W is the weight matrix, and $A^{n}$,${\;A}^{n+1}$ represent the input and output of the n+1 layer, $N_{n+1}$ is the size of A, M is the number of convolution channels, t is the step size, and p and  f are the padding and convolution kernel size. The activation function is usually used after the convolution kernel, in order to make the model fit the training data better and accelerate the convergence, avoid the problem of gradient vanishing, in this article, ReLU is selected as the activation function, as follows:

(3) f(x)=max(0,x)

x is the input value of the upper neural network. the convolutional layer performs feature extraction, and the obtained information is used as the input of the pooling layer. The pooling layer can further filter the information, which not only reduces the dimension of the feature, but also prevents the risk of overfitting. Pool layer generally has average pool and maximum pool. The expression for the pooling layer is as follows [26]:

(4) $$Z_{m}^{n}(i,j)={\left[{\sum_{x=1}^{f}\sum_{y=1}^{f}Z}_{m}^{n}{\left(t*i+x,t*j+y\right)}^{s}\right]}^{\frac{1}{s}}$$

where t is the step size, pixel (i,j) are the same as convolutional layer, s is a specified parameter. When  s=1, the expression is average pooling, and when s→∞, the expression is maximum pooling. *m* is the number of channels in the feature map, *Z* is the output of pooling layer, and the value of s determines whether the output is average pooling or maximum pooling. The other variables have the same meaning as convolution.

After feature extraction and dimensionality reduction of convolutional layer and pooling layer, the fully connected layer maps these features to the labeled sample space. After smoothing, the fully connected layer transforms the feature matrix into one-dimensional feature vector. The full connection layer also contains the weight matrix and parameters. The expression of the full connection layer is as follows:

(5) Y=σ(WI+b)

where I is the input of the fully connected layer, Y is the output, W is the weight matrix, and b is the bias. σ()  is a general term for activation function, which can be softmax, sigmoid, and ReLU, and the common ones are the multi-class softmax function and the two-class sigmoid function.

### 2.2. Fully Convolutional Neural Network

**Figure 2:**
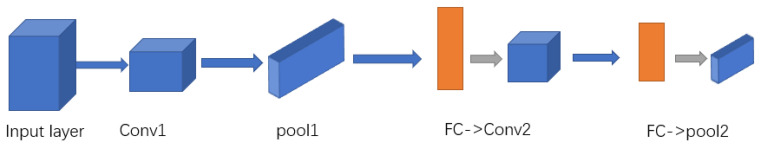
The structure of full convolution model.

In 2015, Long et al. [27] proposed the concept of a fully convolutional neural network, realized the improved segmentation of the PASPA VOC data set (PASCAL Visual Object Classes), and confirmed the feasibility of converting a fully connected layer into a convolutional layer. The difference between fully connected layer and the convolutional layer is that the connections of the neurons in the convolutional layer are only related to a local input region, and the neurons in the convolutional layer can share parameters. This greatly reduces the parameters in the network, improves the calculation efficiency of neural network, and reduces the storage cost. The structure of the full convolution model is shown in Figure 2. 

### 2.3. LSTM Neural Network

**Figure 3:**
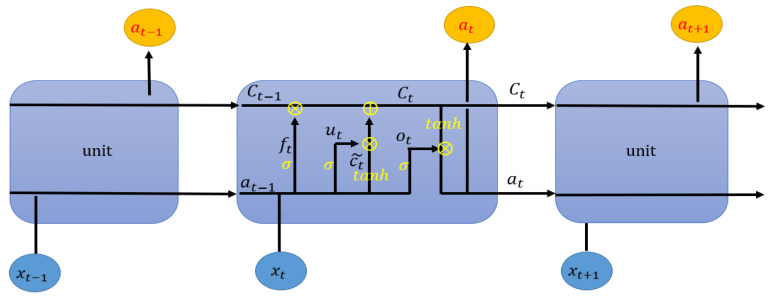
The cell structure of long short-term memory (LSTM).

LSTM (long short term memory) is an improved recurrent neural network (RNN) [28]. LSTM is usually used to deal with time series data. The emergence of LSTM solves the problem of gradient disappearance and gradient explosion in the long-term training process. The output structure of the traditional RNN is composed of bias, activation function, and weight, and the parameters of each time segment are the same. However, LSTM introduces a "gate" mechanism to control the circulation and loss of features. The whole LSTM can be regarded as a memory block, in which there are three “Gates” (input gate, forget gate and output gate) and a memory unit. In LSTM, the order of importance of gate is forget gate, input gate, and output gate. The chain structure of LSTM unit is shown in Figure 3.

(1) Forget gate:

(6) $$f_{t}=\sigma (W_{f}\cdot [a_{t-1},x_{t}]+d_{f})$$

where $f_{t}$ is the forget gate, which means that some features of $C_{t-1}$ are used in the calculation of $C_{t}$. The value range of elements in $\;f_{t}$ is between [0, 1], while the activation function is generally sigmoid, $W_{f}$ is the weight matrix of forgetting gate, $d_{f}$ is the bias, and ⊗ is the gate mechanism, which represents the relational operation of bit multiplication.

(2) Input gate and memory unit update

(7) $$u_{t}=\sigma \left(W_{u}\cdot \left[a_{t-1},x_{t}\right]+d_{u}\right)$$

(8) $$\tilde{C_{t}}=tanh\left(W_{C}\cdot \left[a_{t-1},x_{t}\right]+d_{C}\right)$$

(9) $$C_{t}=f_{t}\circ C_{t-1}+u_{t}\circ \tilde{C_{t}}$$

Among them, $C_{t}$ is the current state of the unit, $C_{t-1}$ is the last state of the unit, and $\tilde{C_{t}}$ represents the update state of the unit, which is obtained from the input data $x_{t}$ and $a_{t-1}$ through the neural network layer. The activation function of the unit state update is *tanh*.$\;W_{u}$ is the weight matrix of the input gate and $\;W_{c}$ is the weight matrix of the output gate.$\;d_{u}$ and $d_{c}$ are bias of the input gate and bias of the output gate, respectively. $u_{t}$ is the input gate, and the element value range is [0,1], which is also calculated by the sigmoid function.

(3) Output gate

(10) $$o_{t}=\sigma \left(W_{o}\cdot \left[a_{t-1},x_{t}\right]+d_{o}\right)$$

(11) $$a_{t}=o_{t}\circ tanh\left(C_{t}\right)$$

Among them, $a_{t}$ is derived from the output gate $o_{t}$ and the cell state $C_{t}$, and the average value of $d_{o}$ is initialized to 1. 

### 2.4. Evaluating Metrics

**Figure 4:**
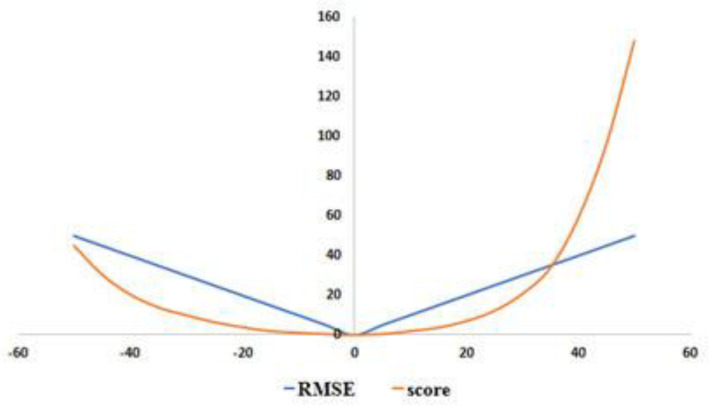
The curve of root mean square error (RMSE) and scoring function (SF).

In order to better evaluate the prediction effect of the model, this article adopts two currently popular metrics for evaluating the RUL prediction of turbofan engines: root mean square error (RMSE) and scoring function (SF), as shown in Figure 4. 

RMSE: it is used to measure the deviation between the observed value and the actual value. It is a common evaluation index for error prediction; RMSE has the same penalty for early prediction and late prediction in terms of prediction. The calculation formula is as follows:

(12) $$RMSE=\sqrt{\frac{1}{X_{n}}\sum_{i=1}^{X_{n}}Y_{i}{}^{2}}$$

where $\;X_{n}$ is the total number of test samples of turbofan engine; $Y_{i}$ refers to the difference between the predicted value pv of RUL of the  i-th turbofan engine and the actual value av of RUL. 

SF: SF is structurally an asymmetric function, which is different from RMSE. It is more inclined to early prediction (in this stage, RUL prediction value pv is less than the actual value  av) rather than late prediction to avoid serious consequences due to delayed prediction. The lower the value of RMSE and SF score, the better the effect of the model. The calculation formula is as follows:

(13) $$score=\{\begin{array}{c} \sum_{i=1}^{x_{n}}e^{\left(\frac{Yi}{13}\right)}-1{,\;Y}_{i}<0;\\ \sum_{i=1}^{x_{n}}e^{\left(\frac{Yi}{10}\right)}-1{,\;Y}_{i}\geq 0; \end{array}$$

In the formula, score represents the score. When RMSE, score, and $Y_{i}\;$ are as small as possible, the effect of the model will be better. 

## 3. 1-FCLCNN-LSTM Prediction Mode

### 3.1. Overall Frameworks

**Figure 5:**
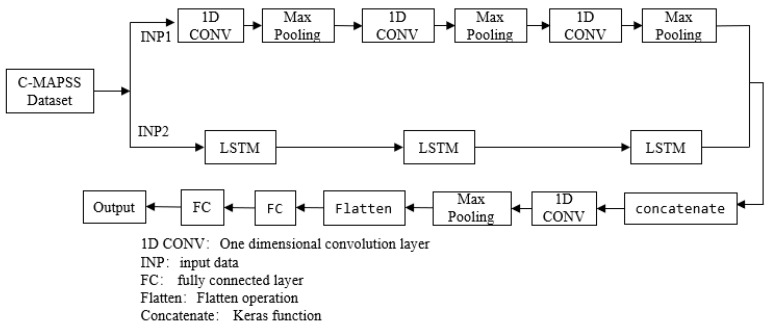
The overall framework of the model.

The life prediction model 1-FCLCNN-LSTM proposed in this paper adopts the idea of classification and parallel processing; 1-FCLCNN and LSTM network extract spatio-temporal features separately, and the two types of feature are fused and then input to the one-dimensional convolutional neural network and fully connected layer. Specifically, first, by preprocessing the C-MAPSS data set, the data are standardized and divided into two input parts: INP1 and INP2. These two parts are input to the 1-FCLCNN and the LSTM neural network. Among them, the 1-FCLCNN is used to extract the spatial feature of the data set. At the same time, the LSTM is adopted to extract the time series feature of the data set. After the feature extraction, the algorithm 1 is applied to fuse the two types of feature and then inputs them into one-dimensional convolutional neural network. Finally, the data through the pooling layer and fully connected layer, is delt with algorithm 2, and the predicted RUL result is obtained. The overall framework of the model is shown in Figure 5.

The feature fusion method, which only splices the feature information without changing the content, preserves the integrity of the feature information, and can be used for multi-feature information fusion. Specifically, in the feature dimension splicing, the number of channels (features) increases after the splicing, but the information under each feature does not increase, and each channel of the splicing maps the corresponding convolution kernel, the expression is as follows:

(14) $$A=\left\{A_{i}|i=1,2,3,\cdots ,channel\right\}$$

(15) $$B=\left\{B_{i}|i=1,2,3,\cdots ,channel\right\}$$

(16) $$D_{\sin gle}=\sum_{i=1}^{channel}A_{i}*K_{i}+\sum_{i=1}^{channel}B_{i}*K_{i+channel}$$

In the formula, the two input channels are A and B respectively, the single output channel to be spliced is $D_{single}$, ∗ means convolution, and K is the convolution kernel. The algorithm of the spatio-temporal feature fusion and RUL prediction in this paper are as follows.
**Algorithm 1** Spatio-temporal Feature Fusion Algorithm
Input: INP1, INP2 Output: Spatio-temporal fusion featureConduct regular processing.Keep the chronological order of the entire sequence, and use one-dimensional convolutional layer to extract local spatial features to obtain spatial information.Extract local spatial extreme values by one-dimensional pooling layer to obtain a multi-dimensional spatiotemporal feature mapLearn the characteristics of the data sequence over time through LSTM.Splice the above two features to get the final spatiotemporal fusion feature.**Algorithm 2** RUL Prediction Algorithm
Input: fusion feature Output:RULInput the fusion feature into one-dimensional convolutional layer for further feature extraction. The convolution kernel contains m filters of size n, each filter learns a single feature, and each column of the output matrix contains a filter weight.Set the maximum pooling layer size to reduce the complexity of output and prevent data from overfitting.Make the multi-dimensional input into one dimension for the transition from the convolutional pooling layer to the fully connected layer.Accelerate the convergence by ReLU function, at this time, each neuron in the first layer is fully connected with the upper node.Reduce the joint adaptability of neurons and improve the generalization ability by the method of the parameter regularization Dropout.Map the learned features to the labeled sample space by the non-linear mapping ability of the fully connected layer network, and the spatio-temporal features are matched with the actual life value to obtain the RUL prediction value.

### 3.2. Model Settings

Compared with multivariate data points composed of single time step sampling, time series data provides more information. In the paper, the sliding window strategy and multivariable time information are adopted to meet the data input requirements of the two network paths. The entire model structure is composed of 1-FCLCNN network, LSTM network, and fully connected layer network. Next are the specific settings of each component.

#### 3.2.1. 1-FCLCNN Network

**Figure 6:**
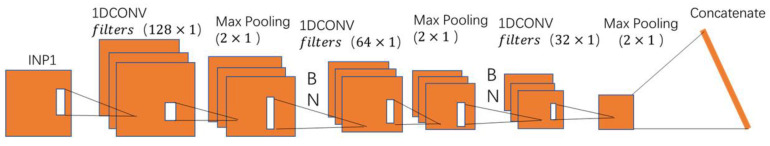
The detailed architecture of the 1-FCLCNN path.

The input of the 1-FCLCNN path is INP1. There are three 1D-CNN layers, which are used to extract the spatial features. The stacked CNN [29] layers are parsed by three max pooling layers. The first 1D-CNN layers consist of 128 filters (filters=128×1). The second consist of 64 filters (filters=64×1), and the third consist of 32 filters (filters=32×1). The convolution kernel size of the three 1D-CNN layers is the same (kernel size=3). “ReLU” activation function(formula 3) is used for the 1D-CNN layers. The Settings of the max pooling layers: pool_size=2, padding="same", strides=2. The batch normalization speeds convergence and controls overfitting after each max pooling layer. The 1-FCLCNN path can be used with or without dropout, making the network less sensitive to initial weights. The detailed architecture is shown in Figure 6. 

#### 3.2.2. LSTM Network

This part is composed of three LSTM layers, which are used to extract time series features of turbofan engines. The input of the LSTM path is INP2. The first LSTM is defined by 128 cell structures, while the second and the third LSTM consist of 64 and 32 cell structures separately. In the LSTM path, the result of each hidden layer is the input of the next layer. Meanwhile, return_sequences=True indicates that the hidden state of output contains the results of total time steps.

#### 3.2.3. Fully Connected Layer

The output of the 1-FCLCNN path is concatenated with the output features of the LSTM path. The resultant vector will be applied as input to the fully connected path. The fully connected path comprises of a convolutional layer, a pooling layer, a flatten layer, and three full connection layers. The convolutional layer consists of 256 filters filters=256×1, the parameter setting of the max pooling layer is consistent with 1-FCLCNN network. Then the data are flattened by flatten layer (transform multidimensional data into one-dimensional data, which are commonly used in the transition from convolutional layer to full connected layer). The first two full connected layers consist of 128 and 32 neurons separately and “ReLU” activation function is utilized. The third layer has 1 neuron as the output to estimate the RUL.

### 3.3. The Process of Model Training

In this paper, the FD001 and FD003 data sets are used. The difference of sensor and engine will cause different physical characteristic state result, therefore, in order to improve the accuracy of the model and the convergence speed of the model, the original monitoring status data are processed by the normalization method. The model limits the data size to between [0, 1].

(17) $$X^{*}{}_{m,n}=\frac{X_{m,n}-X_{n}^{\min}}{X_{n}^{\max}-X_{n}^{\min}},\forall m,n$$

In the formula, $X_{m,n}^{*}$ represents a value of the *m*th data point of the *n*th feature after normalization processing. $X_{m,n}$ represents the initial data before processing. $X_{n}^{max},X_{n}^{min}$ are the maximum and minimum values of the features respectively.

In the model training section, the purpose of training is to minimize the cost function and the loss, and to obtain the best parameters. The cost function as RMSE is defined by the model (Formula (12)). In the meantime, Adam algorithm [30] and Early stopping [31] are adopted to optimize the training process. The Early stopping can not only verify the effect of the model in the training process, but also avoid overfitting. During the training, the normalized data are segmented by sliding window. The input data INP1 and INP2 are in the form of two-dimensional tensors with the size of  ssw×nf, which are processed in parallel paths separately, they are 1D convolutional layer and LSTM network. In order to make the gradient larger and reduce the gradient disappearance problem [32], normalized operation is used after each max pooling layer. In addition, the normalized operation normalizes the activation values of each layer of the neural network to maintain the same distribution. In the meantime, a larger gradient means an increase in convergence and training speed.

The 1-FCLCNN-LSTM training algorithm is as follows:
**Algorithm 3** 1-FCL CNN -LSTM training algorithm
Input: C-MAPSS dataset(FD001, FD003) Output: 1-FCLCNN -LSTM model based on weight determination.Feature selection of data and standardization of data.Prepare training set and test set; normalization processing of training set and test setCalculate the test set label.Extract input data of the network; Use a sliding window to split the data (Define the sliding window function to extract features).Use sliding window to extract training set label and test set label.Use Adam optimization algorithm to update weights. When the number of training periods of 1-FCLCNN -LSTM model is less than the set value or does not reach Early Stopping condition, the extracted features will be input into 1-FCLCNN -LSTM for forward propagation and the predicted output will be obtained. Calculate the error between the predicted value and the actual value.Obtain the trained 1-FCLCNN-LSTM model.


**Figure 7:**
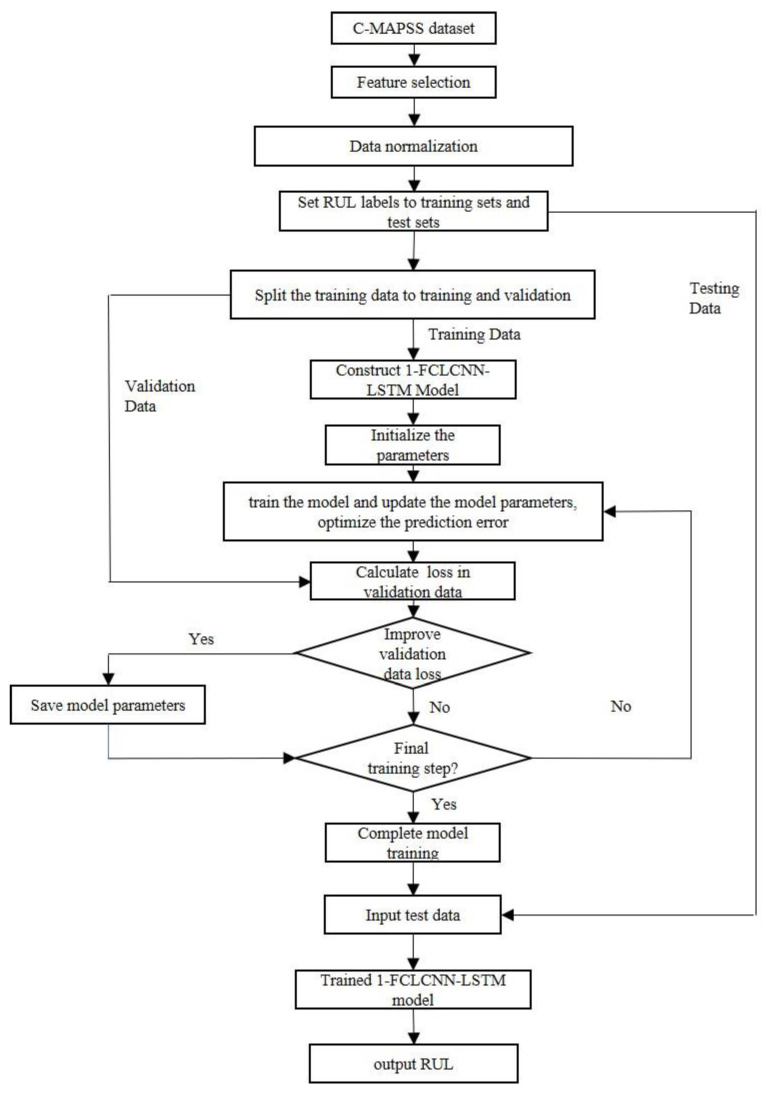
The flow chart of the proposed method.

The overall flow of the proposed method in this paper is shown in Figure 7:

## 4. Experiments and Analysis

First, the C-MAPSS data set is introduced in detail, second, preprocesses the data, test and verify the proposed prediction model on the data set. Then parameter settings are adjusted through the training model. Finally, compares the experimental results with other methods.

### 4.1. C-MAPSS Data Set

**Figure 8:**
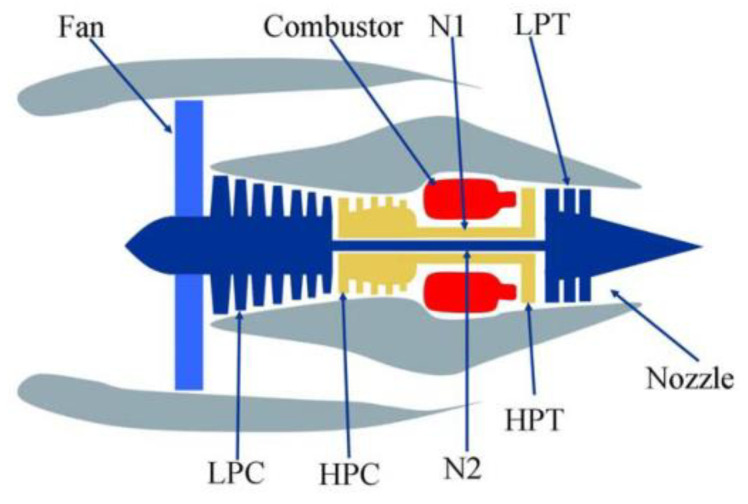
C-MAPSS turbofan engine diagram [34].

In this paper, NASA C-MAPSS turbofan engine degradation data set (https://ti.arc.nasa.gov/tech/dash/groups/pcoe/prognostic-data-repository/) [33] was used, which was derived from C-MAPSS database created by NASA Army Research Lab. The main control system consists of fan controller, regulator, and limiter. Fan controls the normal operation of the flight conditions, sending air into the inner and outer culverts, as shown in Figure 8. A low pressure compressor (LPC) and high pressure compressor (HPC) supply compressed high temperature, high pressure gases to the combustor. Low pressure turbine (LPT) can decelerate and pressurize air to improve the chemical energy conversion efficiency of aviation kerosene. High pressure turbines (HPT) generate mechanical energy by using high temperature and high pressure gas strike turbine blades. Low-pressure rotor (N1), high-pressure rotor (N2), and nozzle guarantee the combustion efficiency of the engine.

The C-MAPSS database contains four subsets of data (FD001-FD004) generated from different time series and including cumulative spatial complexity. Each data subset includes a test data set and a training data set and the number of engines varies in each data subset. Each engine has varying degrees of initial wear-and-tear, and this kind of wear-and-tear is considered normal. There are three operating settings that have a significant impact on engine performance. The engine works normally at the beginning of each time series and fails at the end of the time series. In the training set, the fault increases until the system fails and in the test set, the time series ends at some time before the system fails [35]. In each time series, 21 sensor parameters and 3 other parameters show the running state of the turbofan engine. The data set is provided as a compressed text file. Each row is a snapshot of the data taken during a single operation cycle, and each column is a different variable. The specific contents are shown in Table 1 and Table 2.

According to the needs of experiment, this paper adopts the data set FD001 and FD003 for model verification, and the specific description of the data set is shown in Table 3:

In this table, the training set in the data set includes the data of the entire engine life cycle, while the data trajectory of the test set terminates at some point before failure. FD001 and FD003 were simulated under the same (sea level) condition. FD001 was only tested in the case of HPC degradation, and FD003 was simulated in two fault modes: HPC and fan degradation. The number of sensors and the type of operation parameters are consistent for the four data subsets (FD001-FD004). The data subsets FD001 and FD003 contain actual RUL values, so that the effect of the model can be seen according to the comparison between the actual value and the predicted value. The result of the experiment is to predict the number of remaining running cycles before the failure of the test set, namely RUL.

### 4.2. Data Preprocessing

In the training stage of the model, the original turbofan engine data should be pre-processed, and the pre-processed data can be put into the model to obtain the parameters required by the model. The pre-processing process includes feature selection, data standardization and normalization, setting the size of sliding window, and RUL label setting of training set and test set. The FD001 dataset contains 21 sensor features and 3 operating parameters (flight altitude, Mach number, and throttling parser Angle). The number of running cycles is also one of the features, so with a total of 25 features. In order to ensure the consistency of the input and output of the model and the comparison effect of different data sets, the feature selection of FD003 is consistent with FD001. Because multiple sensors will produce multiple features, in order to eliminate the influence of different dimensions on the prediction results, the normalization method of formula (14) is adopted. The input data are a 2D matrix containing ssw (as the size of the sliding window) with nf (as the number of the selected features). In order to keep the size of input and output of FD001 and FD003 data subsets constant and the data processing process is accelerated. This paper uses a larger window to get more detailed features. The sliding window and the number of features is set to 50 and 25 respectively.

**Figure 9:**
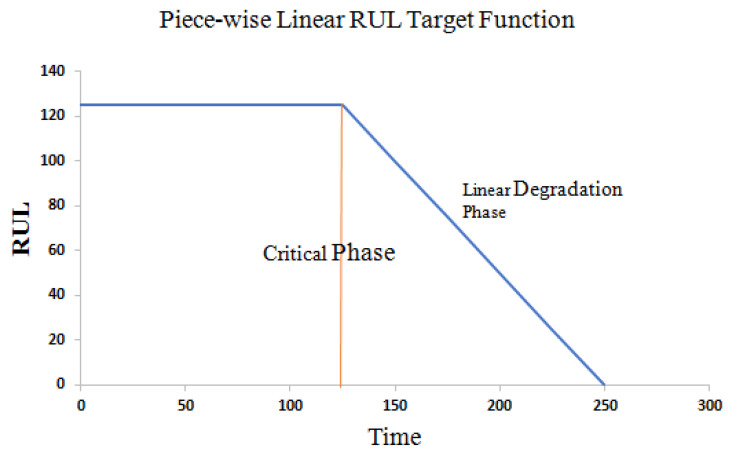
Piece-wise linear remaining useful life (RUL) target function.

In the neural network model, we need to get the corresponding output according to the input data. The state of the system at each time step and the specific information of the target RUL are based on the physical model and are difficult to determine. To solve this problem, different solutions have been proposed. One solution is to simply allocate the required output as the actual time remaining before a functional failure, but at the same time the state of the system will decline linearly [36]. Another option is to obtain the desired output value based on the appropriate degradation model. Referring to the current literature, this paper adopts piece-wise linear degradation model to determine the target RUL [37,38,39,40]. Piece-wise linear regression model can prevent the algorithm from overestimating RUL. For an engine, equipment can be considered healthy during its initial period. The degradation process will be obvious before the whole equipment runs for a period of time or is used by a certain extent, that is, near the end of the life of the equipment. Set the normal working state of the device to a constant value, and the RUL of the device will drop linearly with time after a certain period of time. This model limits the maximum value of RUL, which is determined by the observed data in the degradation stage. The maximum RUL value of the data set observed from the degradation phase of the experiment is set to 125, and the part exceeding 125 is uniformly specified as 125. When the critical period is reached, RUL decreases linearly as the running period increases. The Piece-wise Linear RUL Target Function is shown in Figure 9.

### 4.3. Parameter Settings

**Figure 10:**
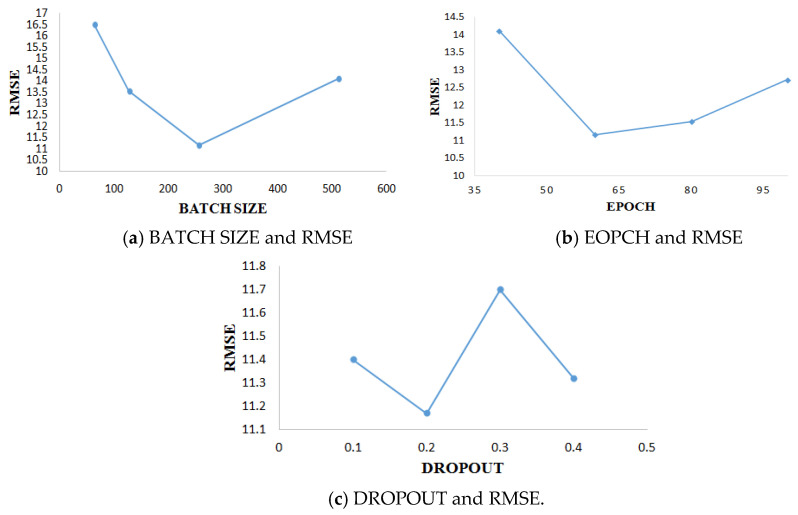
Experimental results of different parameters of FD001.

**Figure 11:**
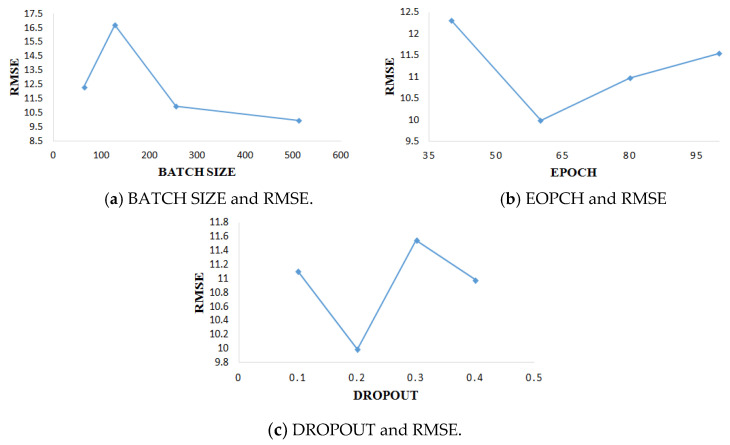
Experimental results of different parameters of FD003.

Because the model needs to adjust parameters in the training process, it is important to select the appropriate parameters for the whole experiment. For two data subsets, FD001 and FD003, each data subset is divided into training set accounting for 85% and verification set accounting for 15%. All data sets are trained in the mini-batch method [35]. For the main parameters involved in the data set in this experiment, this paper adopts a one-by-one optimization method according to the most commonly used values of the parameters. The selection of parameters included epoch (value: 40, 60, 80,100), batch size (value: 64,128,256,512), dropout rate (value: 0.1, 0.2, 0.3, 0.4). In the course of experimental training, if the training error and verification error do not decrease in the five training periods, the training shall be stopped in advance. The parameter results of FD001 are shown in Figure 10. The parameter results of FD003 are shown in Figure 11.

After model training and comparative analysis of experimental results, the parameter setting of FD001 and FD003 data subsets with the best model performance is finally obtained, as shown in Table 4.

### 4.4. Experimental Results and Comparison

**Figure 12:**
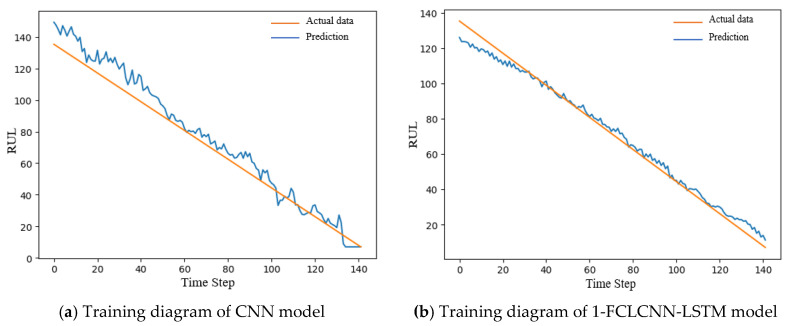
Training diagram of the same FD001 engine under two models.

**Figure 13:**
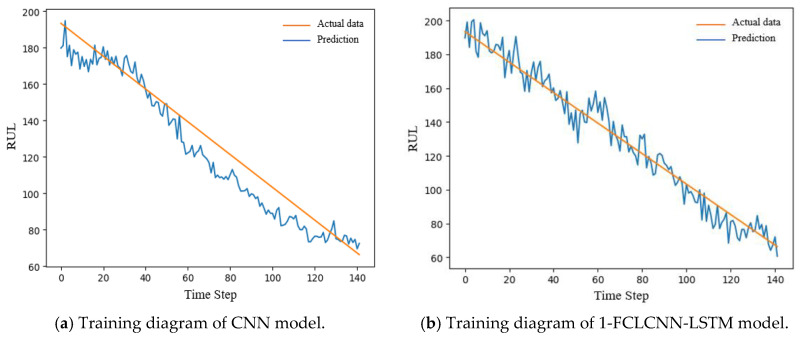
Training diagram of the same FD003 engine under two models.

In this section, we mainly introduce the prediction results of this model and the comparative analysis with the recent popular research methods. With the same data input and the same pretreatment process, the prediction results of the traditional convolutional neural network are compared with the 1-FCLCNN-LSTM model proposed in this paper. The traditional convolutional neural network consists of two convolutional layers, two pooling layers, and a full connected layer. For FD001 and FD003 data subset, this paper compares the training effect of convolutional neural network and FCLCNN-LSTM model under the same data set and engine. The training effects of engines with FD001 and FD003 data subsets on the two models are shown in Figure 12 and Figure 13. The training diagrams of the two models in a single data subset can be obtained as follows: RUL began to decrease with the increase of time step, and finally failed. From the process of RUL reduction, it can be observed that with the increase of time, the higher the prediction accuracy, the closer the predicted value and the actual the values are, which means that the smaller RUL is closer to the potential fault. In this paper, RMSE is used to express the training effect of FD001 and FD003 training sets, as shown in Formula (12). The comparison results are shown in Table 5.

From Table 5 and the training diagrams of the two models on different data sets, it can be concluded that the 1-FCLCNN-LSTM proposed in this paper performs better in the training process than the traditional single CNN neural network. Among them, the RMSE of 1-FCLCNN-LSTM model on FD001 training set was 41% lower than that of CNN model, and the RMSE of 1-FCLCNN-LSTM model on FD003 training set was 46% lower than that of CNN model. FD003 has two fault modes while FD001 has only one, which indicates that the multi-neural network model has certain advantages in dealing with complex fault problems.

**Figure 14:**
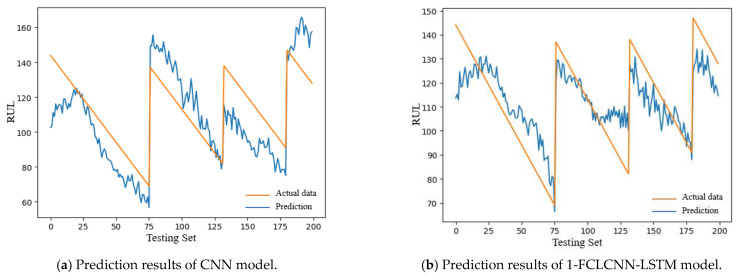
RUL prediction results of FD001 with different models.

**Figure 15:**
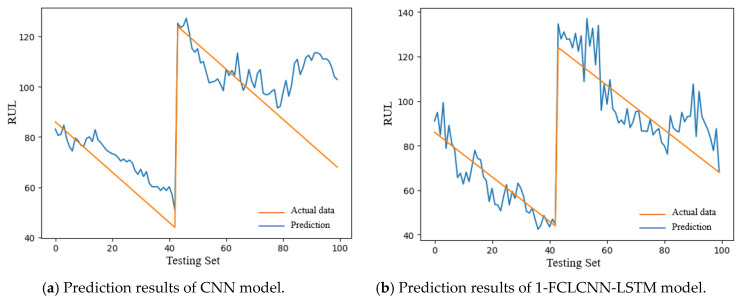
RUL prediction results of FD003 with different models.

The test sets of FD001 and FD003 were input into the trained CNN and 1-FCLCNN-LSTM models to obtain the prediction results, which are shown in Figure 14 and Figure 15, respectively.

In this paper, the RMSE is used to express the effects of FD001 and FD003 test sets, as shown in Formula (12). See Table 6 for details.

It can be seen from Table 6 that the training effect of the model directly affects the performance of the test set of the model. As shown in the above table, the RMSE of 1-FCLCNN-LSTM model on FD001 test set is 35% lower than that of CNN model and the RMSE of 1-FCLCNN-LSTM model on FD003 is 35.5% lower than that of CNN model.

In order to measure the prediction performance of the model more comprehensively, this paper selects the latest advanced RUL prediction method, and compares the deviations of various methods under the same data set. The evaluation indicators are RMSE and the score function, both of which are as low as possible. The comparison results of FD001 data set are shown in Table 7, and the comparison results of FD003 data set are shown in Table 8.

The comparison results with multiple models show that the model proposed in this paper has the lowest score and RMSE values on both FD001 and FD003 data sets. The RMSE of 1-FCLCNN-LSTM model on FD001 was 11.4–36.6% lower than that of RF, DCNN, D-LSTM, and other traditional methods, and the RMSE of 1-FCLCNN-LSTM model on FD003 was 37.5–78% lower than that of GB, SVM, LSTMBS, and other traditional methods. The above results are attributed to the multi-neural network structure and parallel processing of feature information in this model, which can effectively extract RUL information. Compared with the current popular multi-model Autoencoder-BLSTM, VAE-D2GAN, HDNN, and other methods, the RMSE of FD001 was decreased by 4–18%, and the RMSE of FD003 was decreased by 18–37.5% compared with that on HDNN, DCNN, Rulclipper, and other methods. The above results are attributed to the same multi-model structure and multi-network structure, the 1-FCLCNN-LSTM model has advantages in feature processing in the1-FCLCNN path, and the fused data are processed by the 1D full-convolutional layer to obtain more accurate prediction results. The score of 1-FCLCNN-LSTM model in FD001 was 5% lower than the optimal LSTMBS in the previous model. The score of 1-FCLCNN-LSTM model in FD003 was 17.6% lower than the optimal DNN in the previous mode. This indicates that the prediction accuracy of this model in C-MAPSS data set is improved, and no expert knowledge or physical knowledge is required, which can help maintain predictive turbofan engines, as a research direction of mechanical equipment health management.

## 5. Conclusions

This paper has presented a method for RUL prognosis of spatiotemporal feature fusion modeled with 1-FCLCNN and LSTM network. From the current data sets which are issued from some location and state sensors, the proposed method extracts spatiotemporal feature and estimates the trend of the future remaining useful life of the turbofan engine. In addition, for different data sets and prognosis horizon, it is shown that the RUL prognosis error is superior to other methods. Future research will improve the use of the approach for online applications. The main challenge is to incrementally update the prognosis results. The use of the approach for other real applications and large machinery systems will also be considered.

## Figures and Tables

**Table 1:** Column contents of data set file.

Serial NUMBER	Variable Name
1	unit number
2	time, in cycles
3	operational setting 1
4	operational setting 2
5	operational setting 3
6	sensor measurement 1
7	sensor measurement 2
8	…
9	sensor measurement26

**Table 2:** Data description of turbofan engine sensor.

Sensor Number	Sensor Description	Units
1	(Fan inlet temperature)	(°R)
2	(LPC outlet temperature)	(°R)
3	(HPC outlet temperature)	(°R)
4	(LPT outlet temperature)	(°R)
5	(Fan inlet Pressure)	(psia)
6	(bypass-duct pressure)	(psia)
7	(HPC outlet pressure)	(psia)
8	(Physical fan speed)	(rpm)
9	(Physical core speed)	(rpm)
10	(Engine pressure ratio (P50/P2)	——
11	(HPC outlet Static pressure)	(psia)
12	(Ratio of fuel flow to Ps30)	(pps/psia)
13	(Corrected fan speed)	(rpm)
14	(Corrected core speed)	(rpm)
15	(Bypass Ratio)	——
16	(Burner fuel-air ratio)	——
17	(Bleed Enthalpy)	——
18	(Required fan speed)	(rpm)
19	(Required fan conversion speed)	(rpm)
20	(High-pressure turbines Cool air flow)	(lb/s)
21	(Low-pressure turbines Cool air flow)	(lb/s)

**Table 3:** Data sets FD001 and FD003 are detailed.

Data Set	Training Set	Test Set	Operating Conditions	Fault Mode	Number of Sensors	Type of Operating Parameters
FD001	100	100	1	1	21	3
FD003	100	100	1	2	21	3

**Table 4:** Model parameter Settings for FD001 and FD003 data subsets.

	Data Subset	FD001	FD003
Parameter			
epoch		60	60
batch size		256	512
dropout		0.2	0.2

**Table 5:** RMSE training values of FD001 and FD003 under the two models.

	FD001	FD003
CNN	8.25	14.00
1-FCLCNN-LSTM	4.87	7.56

**Table 6:** RMSE predicted by FD001 and FD003 under the two models.

	FD001	FD003
CNN	17.22	15.50
FCLCNN-LSTM	11.17	9.99

**Table 7:** Comparison results of various models on the FD001 dataset.

Models	RMSE	Score
1-FCLCNN-LSTM	11.17	204
HDNN [29]	13.02	245
DCNN [20]	12.61	274
LSTM-FNN [41]	16.14	338
LSTMBS [42]	13.27	216
Autoencoder-BLSTM [23]	13.63	261
VAE-D2GAN [40]	11.60	221
D-LSTM [43]	16.14	338
RF [44]	17.91	480
BiRNN-ED [45]	14.74	273

**Table 8:** Comparison results of various models on the FD003 dataset.

Models	RMSE	Score
1-FCLCNN-LSTM	9.99	234
HDNN [29]	12.22	288
DCNN [20]	12.64	284
LSTMBS [42]	16.00	317
D-LSTM [43]	16.18	852
RF [44]	20.27	711
SVM [44]	46.32	22542
Rulclipper [45]	16.00	317
FADCNN [46]	19.82	1596
GB [44]	16.84	577

## Data Availability

Not applicable.
